# Hermaphroditism Complicated with Extrophy of Bladder

**Published:** 1890-09

**Authors:** Rita Dunlevy

**Affiliations:** New York; Bureau of Surgery, I. H. A.


### HERMAPHRODITISM COMPLICATED WITH EXTROPHY OF THE BLADDER.

RITA DUNLEVY, M. D., NEW YORK.

(Bureau of Surgery, I. H. A.)

Mary H., for so this child was named, was born in Brooklyn, N. Y., in 1873.

When examined the child was found well developed above and below the pelvis, but peculiarly deformed about the pubic region. It was evident that nature had intended to create a man, but for some unknown cause she failed to develop the type of either sex.

The navel and the pubic bones were wanting.

From either groin a sac-like mass grew and hung fully two-thirds the length of the thighs.

At about one-half their length these two sacs coalesced.

Into the upper quadrant of these sacs a portion of the intestine descended, while the lower segment was filled with testicle tissue.

At the angle formed by the junction of these hernial testicle sacs was a mass of erectile tissue about one and a half by two and a half inches, evidently a rudimentary penis.

At times this would erect several inches.

Above this erectile mass was a red mucous surface, the inner coat of the bladder.

From four openings in this bladder the urine dribbled constantly.

In 1881 the case was operated on by Dr. Wm. Tod Helmuth.

He tried by plastic surgery to cover over the bladder, but only succeeded in closing two of the openings.

Later, Dr. Helmuth removed one of the testicle masses and did several other operations with small success.

In May, 1889, the case came before the students of the New York Medical College and Hospital for Women, and was examined, under ether, by Professor Edmund Carleton, M. D.

The following day, in the presence of several members of the profession interested in the case, the faculty, and the students, Professor Carleton operated.

First he removed the remaining testicle from the right sac. Then he denuded the edges of the tissue at upper angle of the bladder and brought the raw surfaces together with several sutures, two of which were hare-lip sutures, the others simple interrupted stitches.

In ten days, when sutures were removed, quite an angle of the opening was found closed in.

On June 16th, again in the presence of members of the profession, faculty, and the students, Professor Carleton operated a second time on the patient.

He removed the mass of erectile tissue, or rudimentary penis, by means of a strong écraseur. There was no hemorrhage following its removal.

Then he denuded the surface above the bladder corresponding to the pubic region, brought the pendant flap of the hernial sacs up over the bladder, and united the two surfaces with twenty-five interrupted silk sutures.

An ivory drainage-tube, devised for the patient by Dr. Wm. Krause, was fitted in the lower left quadrant of the hernial sacs. This to conduct off the urine. The patient was placed in bed in a semi-lateral position, to favor the escape of the urine through the tube.

The remaining portion of the sac was kept constantly supported to prevent the hernia from making traction on the stitches. To guard against bed-sores and give the patient relief, the position had to be changed from time to time.

This rendered it impossible to prevent an escape of urine through and over some of the stitches.

On removal of sutures the parts were found nicely united, except several central sutures.

Their union was prevented by the action of the urine.

June 29th.—Healthy granulation had taken place on the raw edges that failed to unite. This Professor Carleton furthered by skin-grafting.

Meanwhile the patient was fed on the most nourishing diet.

The wound was washed very frequently with calendulated water and kept anointed with calendulated vaseline.

For the first three days the temperature rose to 102° F., then fell to normal and there remained.

As a result of the operation the sac was so reduced in size that the patient could walk, stand, or sit with comfort.

Sensations of a sensual nature, which troubled the patient greatly, ceased entirely.

The great sensitiveness of the parts was removed, and the bladder was well concealed.

July 27th.—The patient was dismissed greatly improved, both mentally and physically, and was delighted with the success of the operation and with the surgeon who had given such relief.

